# Field-Evolved Resistance to Bt Maize by Western Corn Rootworm

**DOI:** 10.1371/journal.pone.0022629

**Published:** 2011-07-29

**Authors:** Aaron J. Gassmann, Jennifer L. Petzold-Maxwell, Ryan S. Keweshan, Mike W. Dunbar

**Affiliations:** Department of Entomology, Iowa State University, Ames, Iowa, United States of America; University of Leeds, United Kingdom

## Abstract

**Background:**

Crops engineered to produce insecticidal toxins derived from the bacterium *Bacillus thuringiensis* (Bt) are planted on millions of hectares annually, reducing the use of conventional insecticides and suppressing pests. However, the evolution of resistance could cut short these benefits. A primary pest targeted by Bt maize in the United States is the western corn rootworm *Diabrotica virgifera virgifera* (Coleoptera: Chrysomelidae).

**Methodology/Principal Findings:**

We report that fields identified by farmers as having severe rootworm feeding injury to Bt maize contained populations of western corn rootworm that displayed significantly higher survival on Cry3Bb1 maize in laboratory bioassays than did western corn rootworm from fields not associated with such feeding injury. In all cases, fields experiencing severe rootworm feeding contained Cry3Bb1 maize. Interviews with farmers indicated that Cry3Bb1 maize had been grown in those fields for at least three consecutive years. There was a significant positive correlation between the number of years Cry3Bb1 maize had been grown in a field and the survival of rootworm populations on Cry3Bb1 maize in bioassays. However, there was no significant correlation among populations for survival on Cry34/35Ab1 maize and Cry3Bb1 maize, suggesting a lack of cross resistance between these Bt toxins.

**Conclusions/Significance:**

This is the first report of field-evolved resistance to a Bt toxin by the western corn rootworm and by any species of Coleoptera. Insufficient planting of refuges and non-recessive inheritance of resistance may have contributed to resistance. These results suggest that improvements in resistance management and a more integrated approach to the use of Bt crops may be necessary.

## Introduction

Transgenic crops engineered to produce insecticidal toxins derived from the bacterium *Bacillus thuringiensis* (Bt) were planted on more than 58 million hectares worldwide in 2010 [1]. Benefits of Bt crops include reduced use of harmful insecticides and regional suppression of some key agricultural pests [2], [3], [4], [5], [6]. Within the United States, and worldwide, more area is planted to Bt maize *Zea mays* L. than any other Bt crop [1]. The western corn rootworm *Diabrotica virgifera virgifera* LeConte (Coleoptera: Chrysomelidae) is among the most serious pests of maize within the United States, with larval feeding on maize roots causing the majority of crop losses from this pest [7]. Beginning in 2003, Bt maize was commercialized for control of western corn rootworm larvae and was rapidly adopted by farmers, constituting over 45% of maize crop in the United States during 2009 [8], [9]. However, the evolution of resistance by the western corn rootworm could cut short the benefits of Bt maize.

The refuge strategy is used in the United States and elsewhere to delay pest resistance to Bt crops [10]. This strategy uses non-Bt host plants as a refuge for Bt susceptible genotypes. Mating of homozygous susceptible pests with pests that are homozygous for Bt resistance produces progeny that are heterozygous for resistance traits. The delay in resistance expected under the refuge strategy becomes greater as the dominance of resistance decreases and is greatest when resistance is completely recessive [11]. Thus, as the area planted to refuge decreases or resistance becomes more dominant, pests are predicted to evolve resistance more quickly [11], [12].

The western corn rootworm has repeatedly demonstrated its ability to adapt to pest management strategies [7]. Examples include the evolution of resistance to conventional insecticides and the cultural practice of crop rotation [13], [14], [15]. The widespread planting of Bt maize targeting western corn rootworm raised concerns that this pest would evolve resistance to Bt. Of additional concern are data suggesting that resistance of western corn rootworm to Bt maize is not recessive [16]. Furthermore, a lack of compliance in planting of refuges has been documented among farmers that grow Bt maize in the United States [17]. Both of these factors are expected to increase the risk of western corn rootworm evolving Bt resistance.

In the present study, we compared populations of western corn rootworm that were sampled from two types of maize fields: problem fields and control fields. Farmers reported severe feeding injury by corn rootworm to Bt maize in problem fields but not in control fields planted to Bt or non-Bt maize. We report that western corn rootworm populations sampled from problem fields showed statistically significant, but not complete, resistance to the Bt maize. Resistance was found only for Cry3Bb1 maize, the type of Bt maize that had been grown historically in those fields, and was not present for maize that produced Bt toxin Cry34/35Ab1. Our results represent the first case of field-evolved resistance by the western corn rootworm to Bt maize and the first case of field-evolved resistance by a coleopteran to Bt toxin, as all previous cases of field-evolved resistance have involved Lepidoptera [18].

## Methods

**Figure 1:**
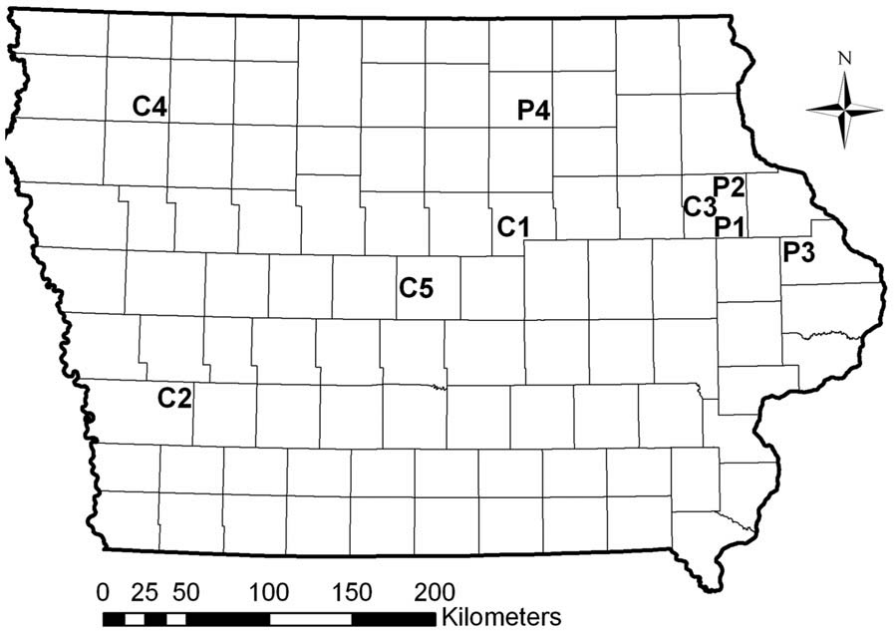
Distribution of sites sampled within Iowa during 2009. Sites beginning with C are control fields and were not associated with feeding injury to Bt maize, and sites beginning with P were problem fields, which were associated with farmer complaints of severe injury to Cry3Bb1 maize by corn rootworm. Codes correspond to Table 1 where a field history is provided along with the corrected survival for these populations of western corn rootworm on Cry3Bb1 maize and Cry34/35Ab1 maize.

Corn rootworm populations were sampled during the summer of 2009 in problem fields and in control fields found within Iowa, USA (Fig. 1). Problem fields were defined as fields with severe feeding injury to Bt maize by corn rootworm and were identified by farmers that contacted the extension service of Iowa State University. Problem fields contained plants that were goose-necked (bent at the plant-soil interface) and lodged (tilted in a pronounced manner), which are characteristic of feeding by corn rootworm larvae. Additionally, farmers noted a high abundance of rootworm adults in problem fields. Upon receiving notification of a problem field, we visited the field and sampled the western corn rootworm present in the field. In all four problem fields, the vast majority of adult *Diabrotica* spp. present in the field were western corn rootworm. In one case a problem field was present on an Iowa State University research farm (Northeast Research and Demonstration Farm; site P4 in Fig. 1 and Table 1). With the exception of this Iowa State University research farm, maize roots were dug from the problem fields to evaluate rootworm feeding injury and the presence of Bt toxin was confirmed by ELISA with a kit (Envirologix, Portland, Maine). Roots were not sampled at random but were selected to confirm the presence of rootworm feeding.

**Table 1:** Sampling date in 2009, corrected survival in bioassays, and history of planting in problem fields (P1–P4) and control fields (C1–C5) from 2003 to 2009.

		Corrected Survival		Field History^a^						
Site	Date Sampled	Cry3Bb1	Cry34/35Ab1	03	04	05	06	07	08	09
P1	11 September	0.61+0.10	0.06±0.04	2	3	3	3	3	3	3
P2	11 September	0.61±0.06	0.03±0.02	2	2	3	5	5	5	5
P3	11 September	0.49±0.05	0.14±0.03	2	2	2	2	3	3	3
P4	14 August	0.40±0.06	0.20±0.10	2	2	2	3	3	3	3
**Mean**		**0.52±0.05**	**0.11±0.06**							
C1	26 September	0.32±0.11	0.25±0.06	2	2	2	3	3	3	3
C2	15 September	0.21±0.08	--------------	1	2	1	2	1	2	2
C3	11 September	0.17±0.05	0.13±0.09	2	2	2	2	2	2	2
C4	23 September	0.10±0.04	0.00±NA	1	2	1	4	2	3	2
C5	01 September	0.06±0.04	0.06±0.05	2	3	1	2	6	1	2
**Mean**		**0.17±0.05**	**0.11±0.06**							

a Field history indicates the crop that was planted in a field each year: 1 = soybean, 2 = maize lacking rootworm active Bt, 3 = Cry3Bb1 maize, 4 = Cry34/35Ab1 maize, 5 = combination of Cry3Bb1 maize and Cry34/35Ab1 maize, 6 = research plots with non-Bt maize and several Bt maize hybrids (mCry3A [46], Cry3Bb1, and Cry34/35Ab1).

Control fields were defined as fields not associated with unexpected feeding by corn rootworm on Bt maize. To allow for comparison with problem fields, only western corn rootworm were sampled from control fields. We sampled five control fields that were widely distributed throughout Iowa. Three of the control fields were located on Iowa State University research farms (C2, C4, C5 in Table 1; Fig. 1). One control field was identified based on a grower complaint of heavy rootworm injury to non-Bt corn (C3 in Table 1; Fig. 1). Another control field (C1) was identified as part of a survey of corn rootworm abundance in Iowa (M. Dunbar pers. obs.). This field was the only control field with a history of Bt maize but there was not apparent rootworm feeding as evidenced by an absence of lodging by maize plants (C1 in Table 1; Fig. 1). Maize roots were not examined in control fields, so the extent of rootworm feeding is unknown.

We interviewed farmers and farm managers to determine cropping history of fields from 2003 to 2009 (Table 1). Individuals were asked if Bt maize had been grown in the field, during which years, and what type of Bt maize (e.g., Cry3Bb1 or Cry34/35Ab1). No questions were asked about planting of refuge, size of refuge, or proximity of the refuge to the Bt field. For years in which Bt maize was not grown in a field, individuals were asked about the type of crop that was grown (e.g., maize or soybeans).

Adult western corn rootworm collected in the field were brought to Iowa State University where they were held in small cages (18 cm×18 cm×18 cm L×W×H) (Megaview Science, Taichung, Taiwan) and provided with food consisting of corn leaf tissue and an artificial diet (western corn rootworm diet, Bio-Serv, Frenchtown, New Jersey). The water source for the adult beetles was 1.5% agar solid, which was 98.5% water by mass, and provided water to the adult western corn rootworm when consumed. Cages were held in an incubator (25°C; 16/8 L/D) and individuals from each population were housed in separate cages. Adults were provided with an oviposition substrate that consisted of moist, finely sieved soil (<180 µm) placed in a 10 cm Petri dish. Eggs obtained from each population were placed separately in 45 mL plastic cups containing moistened sieved soil, and then sealed in a plastic bag and placed in a cold room at 8°C for at least 5 months to break diapause. Following exposure to cold, eggs were stored for one week at 25°C. Eggs were washed from the soil using a screen with 250 µm openings and then placed atop moistened sieved soil held in a 10 cm Petri dish. Neonate larvae began hatching approximately one week thereafter.

Neonate larvae from each population were evaluated in laboratory bioassays for their survival on two transgenic maize hybrids, each of which contained a unique Bt toxin targeting corn rootworm. One hybrid (DeKalb DKC 6169) produced Cry3Bb1. The other hybrid (Mycogen 2T789) produced Cry34/35Ab1. For both of these hybrids, we also evaluated rootworm survival on a near isogenic hybrid that lacked a gene for a rootworm active Bt toxin but otherwise was genetically similar to its respective Bt hybrid. In the case of Cry3Bb1 maize, the non-Bt hybrid was DKC 6172 (DeKalb) and for Cry34/35Ab1 maize the non-Bt hybrid was 2T777 (Mycogen).

Maize plants used in bioassays were grown in a greenhouse (25°C, 16/8 L/D) in 1 L containers made of clear plastic (Reynolds Food Packaging, Shepherdsville, Kentucky) with supplemental lighting provided with 400 W high-pressure sodium bulbs (Ruud Lighting Inc., Racine, Wisconsin). Containers were filled with 750 mL of a 1∶1 ratio of Sunshine Sun Gro SB300 and Sunshine Sun Gro LC1 potting soils (Sun Gro Horticulture Canada Ltd., Vancouver, British Columbia). Seeds were planted one per container at a depth of ca. 4 cm. Beginning two weeks after planting, plants were fertilized weekly with 100 mL of Peters Excel 15-5-15 Cal-Mag Special (Everris International, Geldermalsen, The Netherlands) at a concentration of 4 mg per mL.

Maize seeds of 2T789 and 2T777 were coated with a seed treatment (CruiserMaxx 250, Syngenta, Basel, Switzerland), which contained the neonicotinoid insecticide Thiamethoxam. Prior to planting, this seed treatment was removed by washing ca. 50 seeds in a solution of 1 mL dish detergent (Ultra Palmolive Original, Colgate-Palmolive Company, New York, New York) and 250 mL deionized water. Seeds were placed in the detergent solution for 20 minutes and agitated gently using a stirring plate and magnetic stirring bar. This process was repeated three times with seeds rinsed four times with deionized water between each time they were washed. Seeds were then rinsed four times and allowed to dry for approximately 12 hours, followed by one hour of soaking in a 10% bleach solution, during which they were stirred every 15 minutes. After seeds were removed from the bleach solution, they were rinsed 10 times with deionized water and then allowed to dry for at least 24 hours. This process removed virtually all visible signs of the seed treatment. Insecticidal seed treatment was not applied to DKC 6169 and DKC 6172. However, to ensure that no residual insecticide was present, seeds were bleached following the methods used with 2T789 and 2T777.

Plants were grown in a greenhouse for three to four weeks, until they contained at least five fully formed leaves (V5 stage), and then moved to incubators for bioassays. For bioassays, plants were first trimmed to a height of 20 cm to allow for storage in incubators. Two to three leaves were left on each plant but were trimmed to 8 cm long. Recently hatched larvae (less than 24 hours old) were removed from the soil's surface within their Petri dish using a fine brush and placed at the base of a maize plant on a root that had been exposed by moving away a small amount of soil. Maize plants remained in their original 1 L containers throughout the bioassay. Between 10 and 20 neonates were placed on the base of each plant. Larvae were distributed equally between Bt and non-Bt maize plants. Cups containing plants and larvae were placed in an incubator for 17 days (25°C, 65% RH, 16/8 L/D), and plants were watered as needed.

After 17 days, the aboveground biomass of the plant was excised and the soil, containing roots and larvae, was removed from the 1 L plastic container and placed on a Berlese funnel to extract larvae from the soil. A length of 17 days was selected for bioassays because it allowed sufficient time for some of the fastest developing larvae to reach the third and final instar [19]. Root masses were held on Berlese funnels over 4 days and rootworm larvae were collected in 15 mL glass vials containing 10 mL of 85% ethanol. The average sample sizes per population were 12.7±4.8 (mean ± standard deviation) bioassay cups for Cry3Bb1 maize and for its non-Bt counterpart, and 12.8±4.8 bioassay cups for Cry34/35Ab1 maize and for its non-Bt counterpart. We did not have sufficient western corn rootworm eggs to test one of the populations (C2 from Table 1) on Cry34/35Ab1 maize.

### Data Analysis

Data on the number of field-years (i.e., planting of one field for single year) during which problem fields and control fields were planted to Cry3Bb1 maize were compared using a G test of independence with a Williams's correction [20].

For each bioassay cup, proportional survival was calculated as the quotient of the number of larvae recovered after 17 days divided by the number of neonates initially placed in a bioassay container. The mean proportional survival for each population on each type of maize was analyzed with a two-way, mixed-model analysis of variance (ANOVA) (PROC MIXED in SAS). Data for the two types of Bt maize (Cry3Bb1 maize and Cry34/35Ab1 maize) were analyzed separately. The ANOVA included the fixed factors of field type (problem field vs. control field), maize hybrid (Bt maize vs. non-Bt maize) and their interaction. Random factors in the analysis were population, which was nested within field type, and the interaction between maize hybrid and population nested within field type. Survival data were transformed by the arcsine of the square root to ensure homogeneity of variance and normality of the residuals. Pairwise contrasts were conducted using the PDIFF option in PROC MIXED.

For each population, we calculated corrected survival as the complement of corrected mortality. Corrected mortality was determined using the correction of Abbott [21], and was calculated for each population by adjusting mortality of larvae from each bioassay cup with Bt maize by the average mortality on the non-Bt near isogenic hybrid. Average corrected survival for each population was compared between control fields and problem fields for Cry3Bb1 maize and for Cry34/35Ab1 maize based on a one-way ANOVA (PROC ANOVA in SAS).

Corrected survival also was used to test the significance of three correlations among all populations sampled. We tested for the following correlations: 1) corrected survival of populations on Cry3Bb1 maize and Cry34/35Ab1 maize, 2) corrected survival for populations on Cry3Bb1 maize and the number of years populations had been exposed to Cry3Bb1 maize in the field and 3) corrected survival for populations on Cry34/35Ab1 maize and the number of years populations had been exposed to Cry34/35Ab1 maize in the field. Correlations were measured using a Pearson correlation coefficient and tested for significance against the null hypothesis of ρ = 0 (PROC CORR in SAS).

## Results

The average level of rootworm feeding injury observed in problem fields was 1.8±0.7 nodes (mean ± standard deviation; N = 12) based on the Iowa State University root injury scale, which ranges from 0 nodes (no feeding injury) to 3 nodes (heavy feeding injury) [22]. All roots sampled from problem fields were from maize plants that produced Cry3Bb1 as indicated by ELISA.

Interviews with farmers indicated that problem fields were planted to Cry3Bb1 maize for at least three consecutive growing seasons while only one control field (C1) was planted to Cry3Bb1 maize for any consecutive growing seasons (Table 1). Furthermore, Cry3Bb1 maize was planted for significantly more field-years in problem fields (14 of 28 field-years) than in control fields (6 of 35 field-years) (G = 7.68; df = 1; P = 0.006). By contrast, control fields were planted to a greater diversity of crops and employed an array of management practices to control corn rootworm (Table 1). For example, control fields were planted to soybeans in 7 of 35 fields-years while problem fields were not planted to soybeans in any of the 28 field-years.

**Figure 2:**
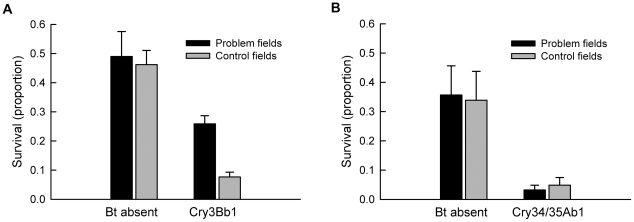
Survival of western corn rootworm on Bt and non-Bt maize. Data are shown for A) Cry3Bb1 maize and B) Cry34/35Ab1 maize. In both cases, survival also is shown for a non-Bt near isogenic hybrid. Bar heights are means and error bars are the standard error of the mean.

Survival in bioassays on Cry3Bb1 maize was affected by a significant interaction between field type and maize hybrid (F = 7.06; df = 1,7; P = 0.03). On the non-Bt hybrid, survival was similar, and did not differ significantly, between populations from problem fields and control fields (P = 0.74) (Fig. 2A). By contrast, on Cry3Bb1 maize, survival was three times higher and significantly greater for insects from problem fields than from control fields (P = 0.011), indicating that insects from problem fields were resistant to Cry3Bb1 maize (Fig. 2A). However, survival was significantly lower on Cry3Bb1 maize than on non-Bt maize for populations from problem fields (P = 0.008), indicating that problem fields contained a mixture of resistant and susceptible individuals, that resistance was incomplete, or that a combination of these factors was present. Additionally, survival was significantly lower on Cry3Bb1 maize compared with non-Bt maize for populations from control fields (P<0.0001).

A different pattern emerged when populations were tested against Cry34/35Ab1 maize and its non-Bt near isogenic hybrid (Fig. 2B). The interaction between field type and maize hybrid was not significant (F = 0.07; df = 1,6; P = 0.80) and the effect of field type was not significant (F = 0.003; df = 1,6; P = 0.96). An effect of hybrid was present, with populations displaying significantly lower larval survival on Cry34/35Ab1 maize than non-Bt maize (F = 61.48; df = 1,6; P = 0.0002) (Fig. 2B). Survival was not significantly different between populations from problem fields and control fields on Cry34/35Ab1 maize (P = 0.95) or on non-Bt maize (P = 0.87). These results indicate that populations were equally susceptible to Cry34/35Ab1 maize, and that this Bt toxin significantly reduced survival.

A second set of complementary analyses were conducted with survival scores for larvae on Bt maize that were corrected for survival on the accompanying non-Bt near isogenic hybrid (Table 1). Corrected survival was significantly higher, and three times greater, for larvae from problems fields than control fields on Cry3Bb1 maize (F = 20.61; df = 1,7; P = 0.003), which indicates that populations from problem fields were resistant to Cry3Bb1 maize. However, no difference between populations from control fields and problem fields was detected on Cry34/35Ab1 maize (F<0.1; df = 1,6; P = 0.99).

**Figure 3:**
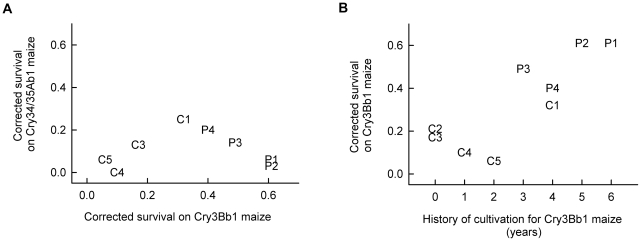
Correlation analysis for corrected survival of western corn rootworm. Correlations are shown for A) survival on Cry3Bb1 maize and Cry34/35Ab1 maize and B) survival on Cry3Bb1 maize and number of years Cry3Bb1 maize was planted in a field. Symbols in the graphs correspond to Table 1, which lists corrected survival for populations on Bt maize and the cultivation history of fields. For (A), no significant correlation was present between survival on Cry3Bb1 maize and Cry34/35Ab1 maize (r = 0.068; df = 6; P = 0.87). For (B), a significant positive correlation was present between corrected survival on Cry3Bb1 maize and the number of years Cry3Bb1 maize had been grown in a field (r = 0.832; df = 7; P = 0.005).

No significant correlation occurred among populations for corrected survival of larvae on Cry3Bb1 maize and Cry34/35Ab1 maize (r = 0.068; df = 6; P = 0.87), indicating an absence of cross resistance between these Bt toxins (Fig. 3A). Cry3Bb1 maize had been grown for at least one field-year in seven of the nine fields sampled (Table 1), and among populations there was a significant positive correlation between the number of years a field had been planted to Cry3Bb1 maize and survival on Cry3Bb1 maize (r = 0.832; df = 7; P = 0.005), indicating that an increased duration of exposure to Cry3Bb1 maize in the field resulted in greater resistance to this Bt toxin (Fig. 3B). Cry34/Cry35Ab1 maize had been grown in only three of the nine fields sampled and for a total of only one field-year alone and five field-years in combination with Cry3Bb1 maize (Table 1). No significant correlation was detected between frequency with which Cry34/35Ab1 maize was cultivated and survival on Cry34/35Ab1 maize (r = −0.56; df = 6; P = 0.15).

## Discussion

Survival of western corn rootworm on Cry3Bb1 maize in laboratory bioassays was significantly higher for insects from problem fields where farmers reported severe root injury to Cry3Bb1 maize than from control fields where such injury was not reported (Fig. 2A). Furthermore, there was a significant correlation between the number of years Cry3Bb1 maize had been grown in a field and survival of western corn rootworm on Cry3Bb1 maize (Fig. 3B). These data indicate that the western corn rootworm is evolving resistance to Cry3Bb1 maize in some populations in Iowa, USA. This is the first case of the western corn rootworm, or any species of beetle, evolving resistance to a Bt toxin in the field [18]. Insects collected from problem fields did not display greater survival on Cry34/35Ab1 maize (Fig. 2B). Additionally, no correlation in survival on Cry3Bb1 maize and Cry34/35Ab1 maize was observed among populations, indicating an absence of cross resistance between these Bt toxins (Fig. 3A).

One factor that may have contributed to the resistance observed here is that Bt maize producing Cry3Bb1 is not considered a high-dose event against corn rootworm [23]. High-dose events are expected to delay resistance by making the inheritance of resistance more recessive [10]. Additionally, genetic analysis of a greenhouse-selected strain found that resistance to Cry3Bb1 maize in western corn rootworm was not a recessive trait [16]. In the context of the refuge strategy, recessive inheritance of resistance caused by high-dose events will reduce survival on Bt crops for heterozygous offspring that result from mating between insects from refuge and Bt fields, thereby delaying resistance [18], [24]. The ability of heterozygous resistant western corn rootworm to survive on Bt maize may have diminished the effectiveness of refuges to delay resistance [12].

A second factor that may have contributed to the evolution of resistance was insufficient refuge populations. Currently, only 50% of Bt maize planted in Midwest complies with US EPA requirements for refuge size and proximity to Bt fields [17]. Insufficient refuge populations also may have contributed to other cases of Bt resistance [25]. In general, larger populations of refuge insects will act to delay pest resistance by decreasing the proportion of homozygous resistant insects in a population, although the magnitude of this effect will depend on the inheritance of resistance, with greater delays occurring for recessively inherited traits [24].

It is noteworthy that our findings are consistent with a greenhouse-selection experiment that found higher survival on Cry3Bb1 maize by western corn rootworm after three generations of selection [16]. In all of the problem fields we studied, Cry3Bb1 maize had been grown for at least three consecutive years (Table 1), which corresponds to three generations of selection in this univoltine pest [7]. Resistance to Cry3Bb1 maize in the greenhouse-selected strain was incomplete, with the selected strain displaying lower fitness on Bt maize than non-Bt maize [16]. In this study, populations from problem fields displayed lower survival on Cry3Bb1 maize than non-Bt maize. This may have been due to resistance in problem fields being incomplete, populations from problem fields containing a mixture of resistant individuals from those fields and susceptible migrants for neighboring fields, or a combination of these two factors.

The landscape-level effects of the resistant populations identified in this study will depend on gene flow, fitness trade-offs that accompany resistance, and selection intensity [26], [27], [28], [29]. Current trends in planting of Bt crops suggest that intense selection for resistance in the field will continue [1]. Fitness costs could act to delay resistance, although the few data currently available suggest that costs of Bt resistance in western corn rootworm may be small [16], [27]. Western corn rootworm appears to have low rates of dispersal, typically traveling less than 40 m per day, although long-distance dispersal is possible [30], [31]. The tendency for short-distance dispersal may help to delay adaptation at a landscape level [29]. Taken together these data suggest that resistance to Cry3Bb1 maize in western corn rootworm should persist and intensify in localized areas, but at a landscape level, some populations may remain susceptible to Cry3Bb1 maize.

It might be the case that the observed resistance by western corn rootworm to Cry3Bb1 maize is a result of pre-adaptation rather than a response to selection. This is unlikely because of the high degree of genetic homogeneity observed among populations of this pest in the Midwest [32], [33]. This genetic similarity is thought to have resulted from the recent and rapid range expansion of the western corn rootworm from the Great Plains to the East Coast [7]. Furthermore, there was a history of selection with Cry3Bb1 maize observed among problem fields (Table 1) and a significant correlation between history of selection in the field and survival on Cry3Bb1 maize in bioassays (Fig. 3b), both of which support the proposition that resistance was the result of selection rather than pre-adaptation.

Recently, Bt maize was commercialized that produces both Cry3Bb1 and Cry34/35Ab1 [34]. Pyramiding of multiple Bt toxins that target the same pest can delay the evolution of resistance to either toxin when most individuals that are resistant to one toxin are killed by the other toxin [35]. In populations where western corn rootworm populations have begun adapting to Cry3Bb1, the benefit of pyramiding two Bt toxins may be diminished [36]. However, the lack of cross resistance between these toxins (Fig. 3a) suggests that pyramiding Cry3Bb1 with Cry34/35Ab1 may still act to delay resistance in problem fields at least as long as, if not longer than, the cultivation of maize producing only a single toxin.

Although no cases of field-evolved resistance were reported during the first decade of commercialization for Bt crops, several recent cases have been reported [12], [25], [37], [38], [39], [40]. Typically, there is a lag between the introduction of an insecticide and the first occurrence of resistance, which is then followed by a steady increase in the cumulative number of occurrences [41]; a trend that would clearly be undesirable for Bt crops. Stern et al. [42] outlined the foundation of integrated pest management by advocating the application of multiple methods to control pest populations, thus delaying or avoiding problems that include, but are not limited to, pest resistance [43]. To date, the widespread planting of Bt crops has resulted in pest resistance for only a small subset of all pest populations managed by this technology [18]. However, these recent cases suggest a need to develop more integrated management solutions for pests targeted by Bt crops [44], [45]. A common pattern observed among problem fields in this study was the consecutive planting of the same type of Bt maize over several seasons (Table 1). Even with resistance management plans in place, sole reliance on Bt crops for management of agriculture pests will likely hasten the evolution of resistance in some cases, thereby diminishing the benefits that these crops provide.
